# Mapping the Use of Artificial Intelligence–Based Image Analysis for Clinical Decision‐Making in Dentistry: A Scoping Review

**DOI:** 10.1002/cre2.70035

**Published:** 2024-11-26

**Authors:** Wei Chen, Monisha Dhawan, Jonathan Liu, Damie Ing, Kruti Mehta, Daniel Tran, Daniel Lawrence, Max Ganhewa, Nicola Cirillo

**Affiliations:** ^1^ Melbourne Dental School The University of Melbourne Carlton Victoria Australia; ^2^ CoTreatAI, CoTreat Pty Ltd. Melbourne Victoria Australia

**Keywords:** artificial intelligence, convolutional neural networks, dentistry, image analysis

## Abstract

**Objectives:**

Artificial intelligence (AI) is an emerging field in dentistry. AI is gradually being integrated into dentistry to improve clinical dental practice. The aims of this scoping review were to investigate the application of AI in image analysis for decision‐making in clinical dentistry and identify trends and research gaps in the current literature.

**Material and Methods:**

This review followed the guidelines provided by the Preferred Reporting Items for Systematic Reviews and Meta‐Analyses Extension for Scoping Reviews (PRISMA‐ScR). An electronic literature search was performed through PubMed and Scopus. After removing duplicates, a preliminary screening based on titles and abstracts was performed. A full‐text review and analysis were performed according to predefined inclusion criteria, and data were extracted from eligible articles.

**Results:**

Of the 1334 articles returned, 276 met the inclusion criteria (consisting of 601,122 images in total) and were included in the qualitative synthesis. Most of the included studies utilized convolutional neural networks (CNNs) on dental radiographs such as orthopantomograms (OPGs) and intraoral radiographs (bitewings and periapicals). AI was applied across all fields of dentistry ‐ particularly oral medicine, oral surgery, and orthodontics ‐ for direct clinical inference and segmentation. AI‐based image analysis was use in several components of the clinical decision‐making process, including diagnosis, detection or classification, prediction, and management.

**Conclusions:**

A variety of machine learning and deep learning techniques are being used for dental image analysis to assist clinicians in making accurate diagnoses and choosing appropriate interventions in a timely manner.

## 1 Introduction

In the modern era of rapid technological development, artificial intelligence (AI) has begun to permeate nearly every field of work. At its core, AI aspires to mimic the human brain, identifying patterns and understanding relationships between attributes and variables. Developments in AI have given rise to well‐established models that can exemplify traits such as learning, critical thinking, and decision‐making (Lingam et al. 2022). The most general and superficial classification under the broader AI umbrella term is shallow machine learning (Janiesch, Zschech, and Heinrich 2021). Algorithms in this classification are derived from decision trees or statistical models, which commonly involve linear and logistic regressions, Naive Bayes, and clustering. Taking a deeper step into the realm of machine learning, there is the introduction of artificial neural networks (ANNs). The networks consist of one or two layers of processing units, similar to artificial neurons. These neural units interact and continuously adjust as the network is trained and refined by researchers inputting data sets. In contrast, deep learning consists of many hidden layers embedded within the network architecture between the input and output levels. The additional depth allows for advanced operations and a greater capability to grasp complex patterns and intricacies. Among the frontiers of deep learning AI are convolutional neural networks (CNNs) and recurrent neural networks (RNNs). Ultimately, AI has an innate reliance on three major components: the computing capacity of hardware, the capability of algorithmic software, and an abundance of adequate data input (Ding et al. 2023).

Even fields distant from the forefront of computer sciences can reap the benefits, as they ride the tailwinds of constant digital advancements. To no exception, AI's integration into the field of dentistry already shows exciting improvements to current clinical practice (Ding et al. 2023). Deep learning has been applied to various facets of clinical care spanning disease detection, classification, prediction, prognosis, and management. Its integration as a clinical adjunct holds tremendous promise for advancing the goals of contemporary dentistry. Modern dentistry pillars include minimal intervention and nominalistic approaches in alignment with evidence‐supported treatment decisions to maximize long‐term oral health (Carounanidy and Sathyanarayanan 2009; Jingarwar, Bajwa, and Pathak 2014). Currently, AI's impact already extends to a wide array of dental specialties, ranging from interproximal caries detection in cariology (Bayraktar and Ayan 2022; García‐Cañas et al. 2023) to three‐dimensional jaw visualization and reconstruction for treatment planning in oral and maxillofacial surgery (OMFS) (Mima et al. 2022; Xu, Liu, and Zheng 2019).

Amidst the rapidly evolving landscape of AI technology, a multitude of proposed applications and algorithms have emerged. Therefore, the objective of this scoping review is to provide a comprehensive assessment and summary of the existing literature on AI‐based image analysis and its impact on decision‐making in clinical dentistry. This review aims to scope and illustrate the variety of existing models, their current utilization among different imaging modalities, research gaps, and emerging trends and frontiers within this applied discipline.

## 2 Methods

### 2.1 Search Strategy

This scoping review was conducted in accordance with the Preferred Reporting Items for Systematic Reviews and Meta‐Analyses Extension for Scoping Reviews (PRISMA‐ScR) guidelines (Tricco et al. 2018), as reported in Table S1. The following databases were searched:

1. PubMed
2. Scopus

Studies published before the month of June 2023 were included when searching databases, with no restrictions placed on the date of publication. Studies that were not published in English, studied non‐human populations, and all reviews were excluded via automatic database filtering tools. Furthermore, conference papers and non‐peer‐reviewed literature were manually excluded by reviewers.

The aim of this scoping review was to explore the incorporation of AI‐based image analysis into clinical decision‐making within dentistry. To obtain a broad initial range of studies, synonyms for, or terms associated with AI were included in the search string such as “convolution neural network,” “machine learning,” “deep learning,” and “support vector machine.” To focus our search strategy on image analysis, keywords such as “diagnosis” and “accuracy” were included; however, classification into specific imaging modalities was performed at a later screening stage.

The following search string was utilized to obtain the initial set of relevant studies on PubMed: ((“Artificial Intelligence”) OR (“AI”) OR (“Artificial neural network”) OR (“Conventional neural network”) OR (“Convolutional neural network”) OR (“Deep learning”) OR (“Machine learning”) OR (“Computational Intelligence”) OR (“Machine Intelligence”) OR (“Support vector machine”) OR (“Artificial Intelligence”[Mesh]) OR (“Deep Learning”[Mesh])) AND ((Dent*[Title/Abstract]) OR (Dentistry[Mesh]) OR (“teeth”) or (“tooth”) OR (“oral”)) AND ((“Diagnos*”) OR (“Decision‐making”) OR (“Computer‐aided diagnosis”) OR (“Clinical Decision‐Making” [Mesh]) OR (“Decision Making, Computer‐Assisted” [Mesh])) AND ((“Efficacy”) OR (“Accuracy”) OR (“Effectiveness”)). This search was modified when searching Scopus with the additional keyword combination: AND (EXCLUDE (DOCTYPE, “re”)) AND (LIMIT‐TO (LANGUAGE, “English”)) AND (LIMIT‐TO (EXACTKEYWORD, “Human”)).

To maintain a broad scope, studies in all fields and specialities of clinical dentistry (excluding forensic and teledentistry) were considered in this study. Reviewers cross‐checked and manually removed studies deemed irrelevant based on the AI's role in decision‐making, field of application and imaging modality.

### 2.2 Data Extraction

Relevant papers obtained from both databases were compiled, and duplicates were removed. The remaining papers were then divided between three pairs of reviewers and screened by titles and abstracts on the screening website Rayyan (https://www.rayyan.ai/). Studies were classified by dental field, imaging modality, and application of AI and included/excluded based on these categories.

Once all conflicts between reviewers were resolved via team discussion, the remaining included papers were extracted and imported into a data extraction table, where studies were categorized based on imaging modality, field of dentistry, AI's role in decision‐making, method of AI application, and type and classification of AI algorithms used. The role of AI in decision‐making was separated into three main categories: diagnosis/detection/classification, prediction, and management. In terms of AI application, algorithms with a direct clinical influence on decision‐making or those responsible for segmentation were included, whereas studies with AI utilized solely as a research technique with no clinical influence were excluded. Studies in all fields of dentistry were included, except for forensic dentistry, as these findings have more limited application in general clinical dentistry and the treatment of living patients.

Additional exclusion criteria included histology, in vitro methods, traditional Chinese medicine, and teledentistry, as these were deemed irrelevant to the “chairside” clinical dentistry focus of this review. Studies that involved no imaging input at all or utilized forms of imaging uncommon in clinical dental practice were also excluded. Additionally, any non‐peer‐reviewed papers or irretrievable full‐text studies were excluded (Table S2).

### 2.3 Data Charting

Once the final set of included studies was established, data collected from the data extraction table were categorized and presented in graphical form. The graphs illustrate the distribution of studies across various categories, including the role in decision‐making, dental field, application of AI, imaging modality, and AI algorithm. Additional graphs were included to display and compare the prevalence of imaging modalities within each dental field.

## 3 Results

### 3.1 Selection of Sources of Evidence

**Figure 1:**
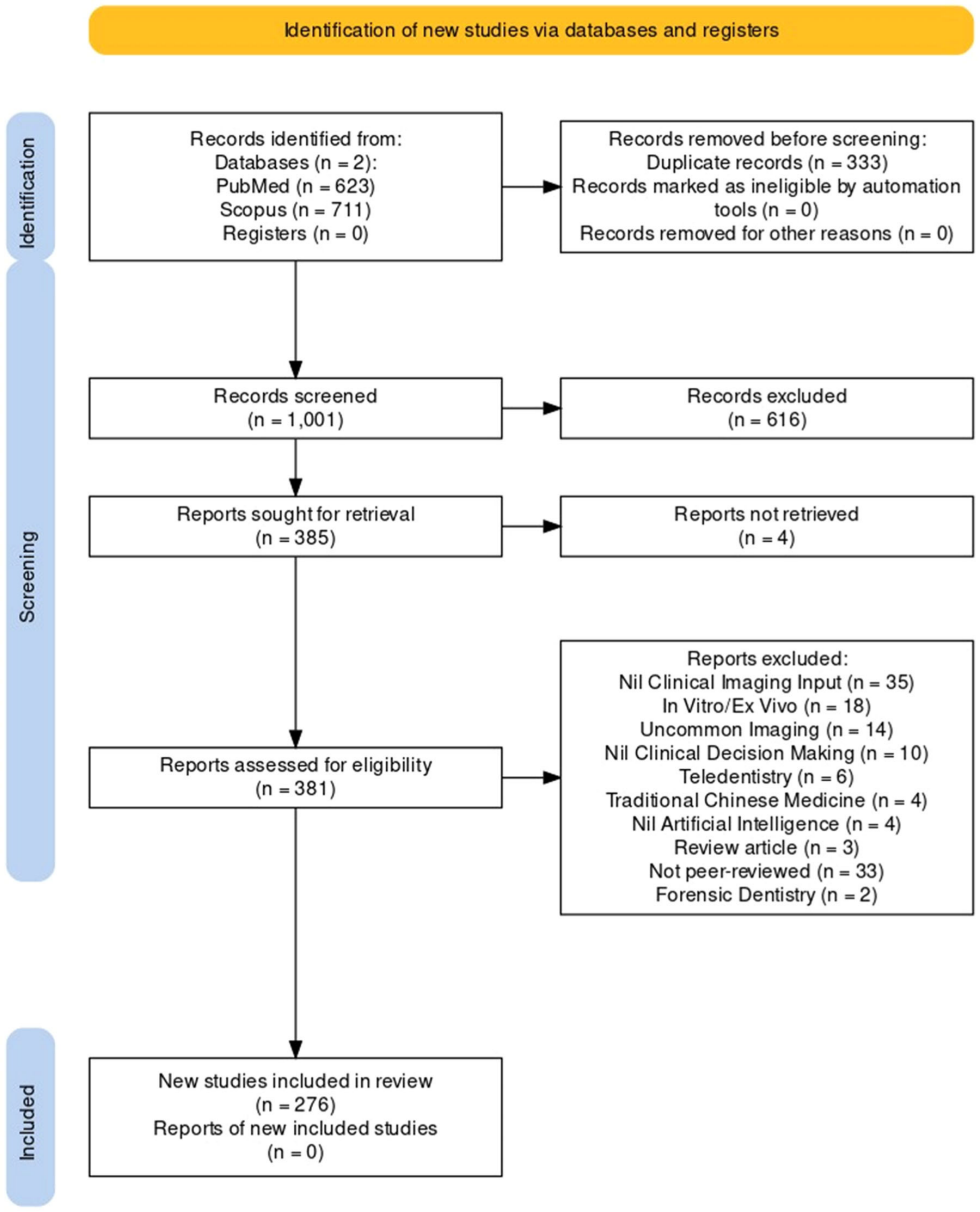
Preferred Reporting Items for Systematic Reviews and Meta‐Analyses Extension for Scoping Reviews (PRISMA‐ScR) flowchart.

The PRISMA flowchart of the scoping review process is shown in Figure 1. The search strategy retrieved 1334 articles from two databases (PubMed and Scopus). Following the removal of 333 duplicates, title and abstract screening of 1001 articles was conducted. Full‐text screening of 381 articles was completed, of which 109 articles were excluded for reasons outlined in Figure 1. Finally, 276 articles were included in the qualitative synthesis. A summary of the relevant data from the included articles is presented in Table S1.

### 3.2 Characteristics of Sources of Evidence

The majority of the included articles were published from 2019 onward, with the number of publications increasing each year (Figure S1). The sizes of the data sets ranged from 10 to 83,676 images, totaling 601,122 items.

#### 3.2.1 Application of AI in Clinical Decision‐Making

The majority of articles discussed the use of AI to directly influence clinical decision‐making (*n* = 231, 84%), whereas some involved AI for segmentation of anatomical structures (*n* = 45, 16.3%) (Figure S2). AI‐based image analysis was most commonly used for diagnosis, detection, or classification (*n* = 233, 84.4%), followed by prediction (*n* = 30, 10.9%) and management (*n* = 13, 4.7%) (Figure S3).

**Figure 2:**
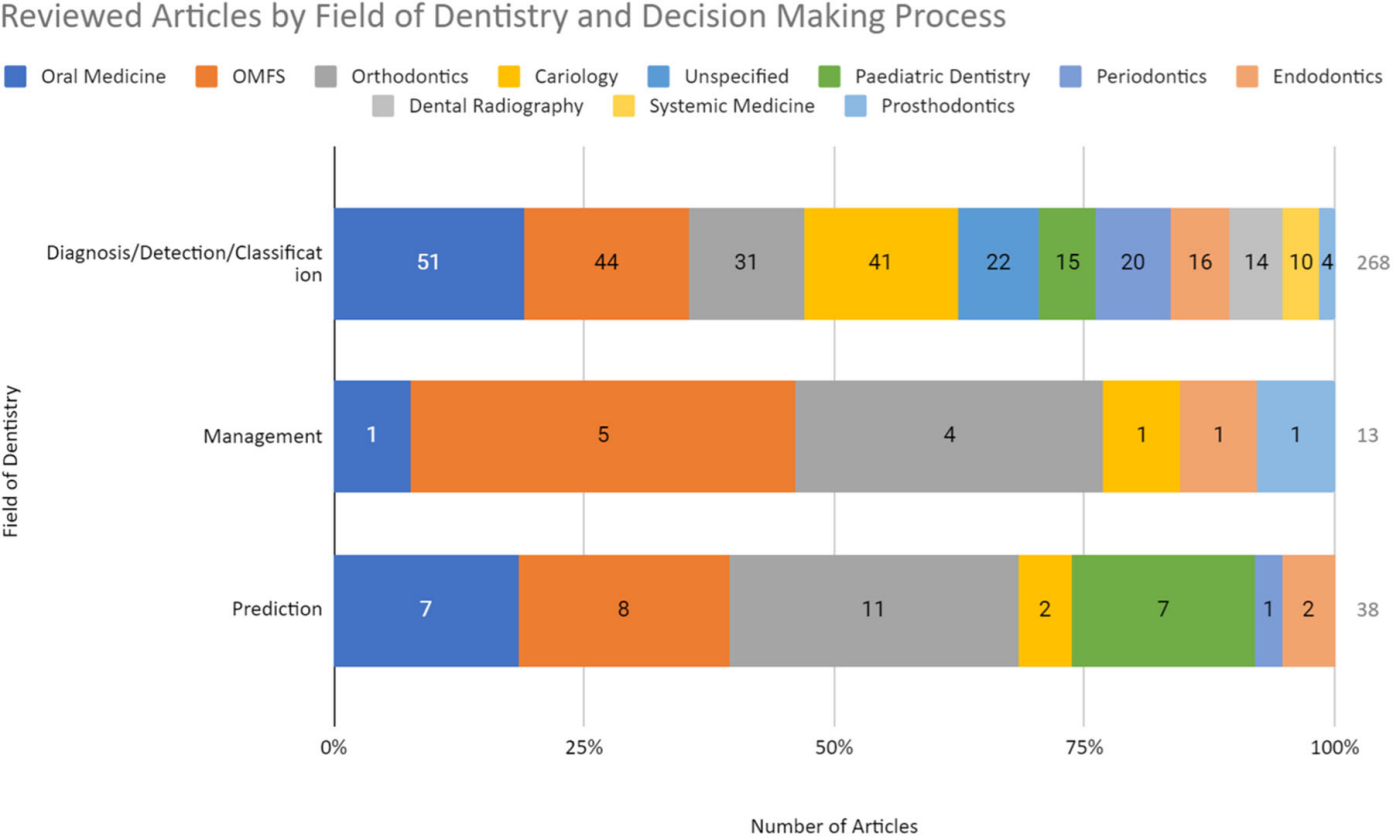
AI‐assisted decision‐making process and fields of dentistry involved in the reviewed articles. Many articles discussed the use of AI in multiple fields and were categorized as such; therefore, there is a discrepancy in the total number. OMFS, oral and maxillofacial surgery. Cariology (operative dentistry).

Many articles discussed the use of AI across multiple fields of dentistry and hence were categorized into more than one area (*n* = 32, 12%). Of the 268 articles that evaluated AI application in diagnostic decision‐making, the greatest proportion was applied in the fields of oral medicine (*n* = 51, 19%), followed by OMFS (*n* = 44, 16%) and operative dentistry (*n* = 41, 15%) (Figure 2). Out of the 13 articles that assessed AI application in management decision‐making, the greatest proportion was applied in the fields of OMFS (*n* = 5, 38%), followed by orthodontics (*n* = 4, 31%) (Figure 2). From the 30 articles that analyzed predictive decision‐making with AI, the most common field of application was orthodontics (*n* = 11, 29%), followed by OMFS (*n* = 8, 21%) and oral medicine (*n* = 7, 18%) (Figure 2).

**Figure 3:**
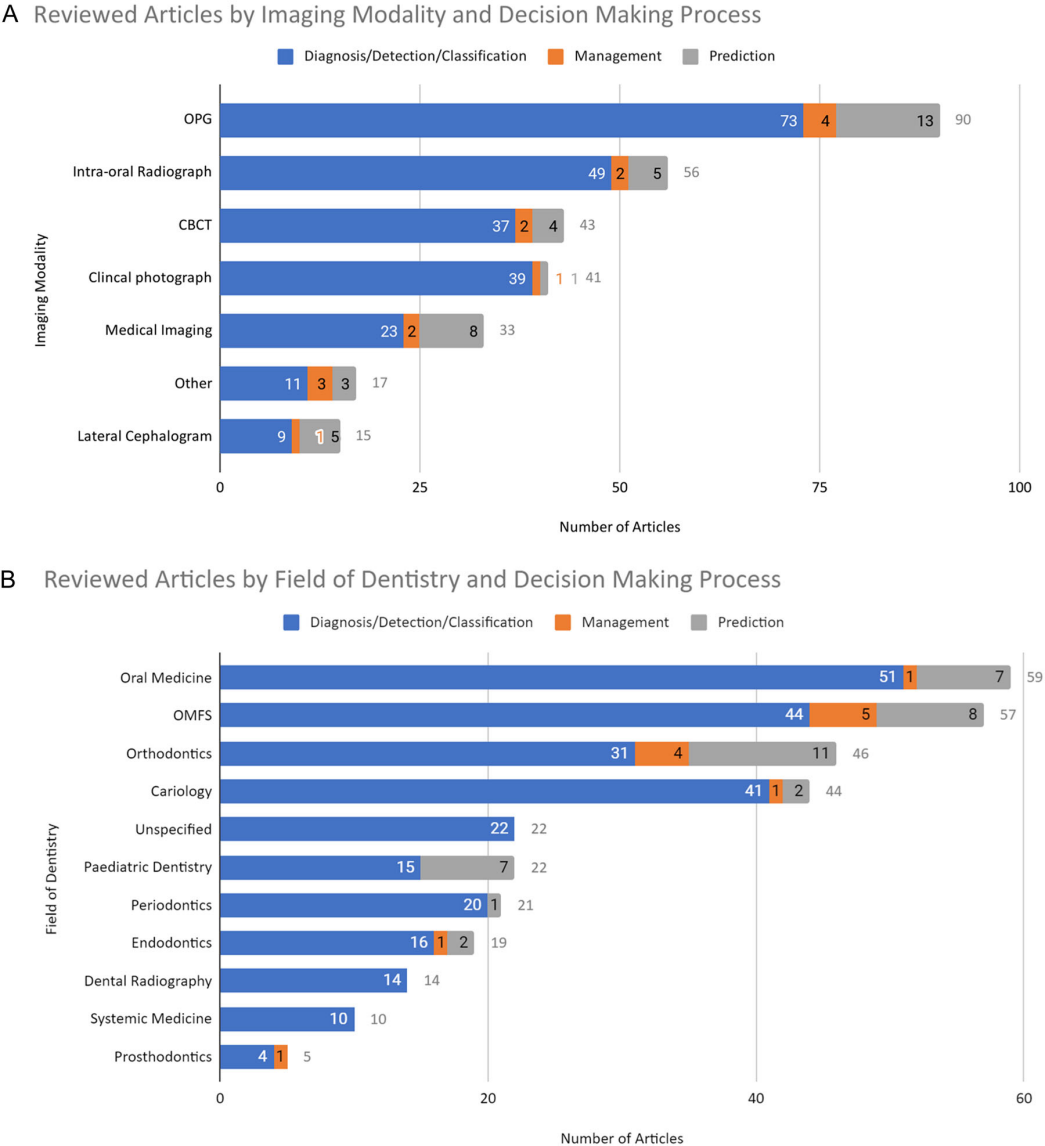
(A) The distribution of reviewed articles by imaging modality utilized as the input for AI analysis and decision‐making process. Abbreviations: CBCT, cone‐beam computed tomography; OPG, orthopantomogram. Medical imaging includes magnetic resonance imaging (MRI), computed tomography (CT), and ultrasound. Others include optical coherence tomography (OCT), hand‐wrist radiograph, phone images, video analysis, digital infrared thermal imaging, and 3D dental scanner. (B) The distribution of reviewed articles by the field of dentistry and the decision‐making process involved. OMFS, oral and maxillofacial surgery.

Similar to articles exploring AI applications in more than one field of dentistry, many models utilized more than a single imaging modality (*n* = 17, 6%). The input imaging modality most commonly evaluated as the input across all decision‐making categories was orthopantomograms (OPGs)—30% of diagnostic articles (*n* = 73), 27% of management articles (*n* = 4), and 33% of prediction‐based articles (*n* = 13) (Figure 3). Following OPG, AI‐assisted diagnostic decision‐making was assessed with intra‐oral radiographs (*n* = 49, 20%) and management and predictive‐based decision‐making with cephalograms (*n* = 1 and *n* = 5, respectively) (Figure 3A).

#### 3.2.2 Fields of Dentistry

AI‐based image analysis was applied most commonly in the following fields of dentistry: oral medicine (*n* = 59, 19%), OMFS (*n* = 57, 16%), orthodontics (*n* = 46, 12%), and operative dentistry (*n* = 44, 15%) (Figure 3B).

The field of dentistry with the greatest proportion of articles reviewing AI‐assisted diagnostic decision‐making was for the detection of carious lesions (*n* = 41, 93%). Prosthodontics was the field with the greatest proportion of articles evaluating AI for management decisions (*n* = 1, 20%) (Figure 3B). Notably, the field with the highest proportion of AI applications in predictive decisions was pediatric dentistry (*n* = 7, 32%) where AI was used for dental age estimation and orthodontics (*n* = 11, 24%) for the prediction of growth and development using cephalograms.

Eighty‐six percent of oral medicine–related articles (*n* = 51) discussed AI for diagnostic decision‐making, particularly for the detection of oral cancer and associated lymph node metastases and odontogenic cystic lesions. Within the OMFS field (*n* = 57) for the detection of anatomical structures (*n* = 44) (Figure 3B), AI was most commonly used for the detection of impacted third molars and their proximity to the inferior alveolar nerve, or the detection of mandibular fractures.

#### 3.2.3 Imaging Modalities

Imaging modalities most commonly used for AI analysis were OPGs (*n* = 90, 33%), intra‐oral radiographs (*n* = 56, 20%), cone‐beam computed tomography (CBCTs) (*n* = 43, 15%), and clinical photographs (*n* = 41, 14%) (Figure 3A). The imaging modality with the greatest proportion of articles assessing AI‐assisted diagnostic decisions was clinical photographs (*n* = 39, 95%) for the detection of carious lesions and OSCC, whereas cephalogram was the imaging modality with the greatest proportion of prediction‐based articles (*n* = 5, 33%).

#### 3.2.4 AI Algorithm

**Figure 4:**
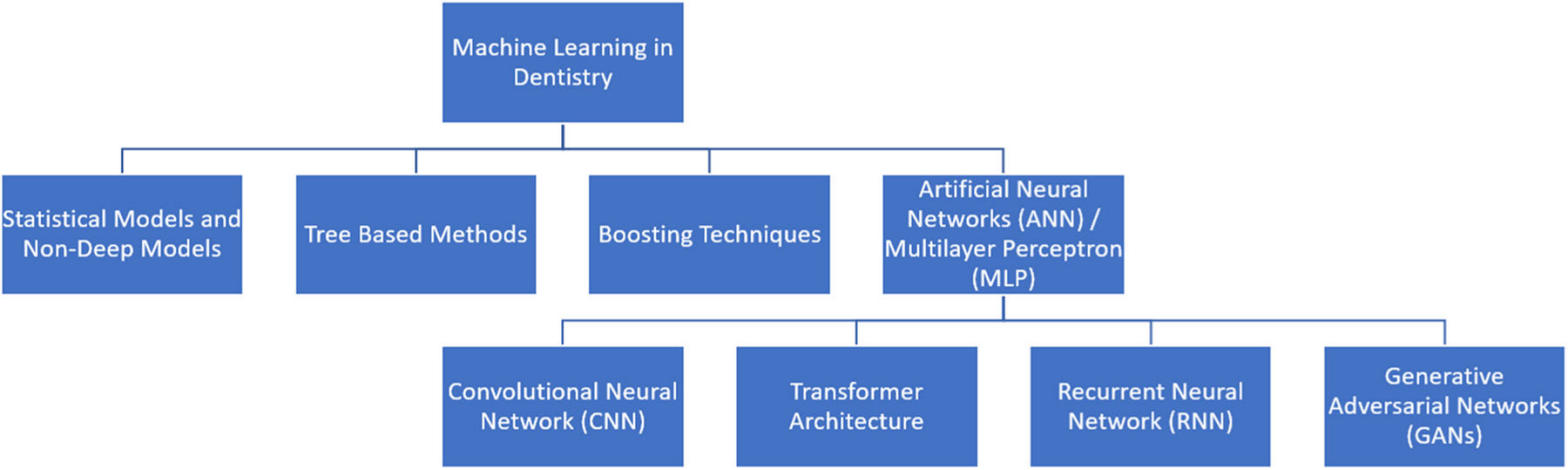
The classification of machine learning algorithms demonstrated in the reviewed articles.

Figure 4 demonstrates the classification of AI models assessed in the reviewed articles. The majority of articles demonstrated the use of ANN/multilayer perceptron (MLP) (*n* = 258, 93%) (further details in Figure S4) to address clinical problems, of which the majority used CNN models (*n* = 204, 79%) (further details in Figure S5). The most commonly utilized CNN model was ResNet (*n* = 55, 27%), followed by U‐net (*n* = 31, 15%) and R‐CNN (*n* = 31, 15%) (Figure S6).

#### 3.2.5 Summary of Largest Data sets

Hernandez et al. (2021) conducted a study with the largest data set to date, comprising 83,676 head and neck CT slices from 549 patients. Their CNN system achieved high accuracy in detecting dental artifacts on a slice‐by‐slice basis, demonstrating the potential use of AI in improving diagnostic precision. Similarly, Kearney et al. (2022) utilized a large data set of 80,326 images for training, with images for validation and testing, to develop a tool for determining clinical attachment loss (CAL) in the diagnosis of periodontal disease. Their model accurately calculated CAL within 1 mm of clinician‐determined standards, highlighting its clinical relevance. Additionally, Fu et al. (2020) developed a CNN model using 44,409 images, with both external and internal validation, and found that the model's performance in detecting oral cancer was comparable to that of oral cancer specialists. Collectively, across all 276 articles included in this qualitative synthesis, approximately 600,000 images were used in various phases of training AI systems, validation processes, and testing.

## 4 Discussion

In this scoping review, we systematically examined the implementation of AI‐based image analysis for clinical decision‐making in dentistry. The review sought to provide a comprehensive overview of the current research present in this rapidly emerging field by analyzing a diverse range of studies. Next, we summarized the main themes and trends that emerged from the literature, address existing research gaps, and consider the potential implications of AI‐based image analysis for dental practitioners in clinical decision‐making.

### 4.1 Summary of Evidence

This review identified 276 primary studies addressing AI‐based image analysis, with the majority of the articles emerging after 2019. This reflects the rapidly evolving nature of AI in an attempt to aid clinical decision‐making.

The most frequently researched categories were oral medicine, OMFS, and operative dentistry, where the technology was used for neoplastic disease, anatomical and structural interpretations, and detection of caries. For all fields of dentistry, the specific use of AI has been primarily in the detection/diagnosis/classification. This is likely due to improving the efficiency of identifying issues so that dental clinicians can act promptly to address them for patients. One such example is in oral medicine where it can be used to identify cancers and metastatic disease, which necessitates early identification and treatment. Currently, such diagnoses require specialist input; however, with such technology, it may assist general dentists to flag findings and expedite the treatment pathway for patients. Interestingly, the greatest proportion of predictive AI studies was in orthodontics (*n* = 8, 21%) and OMFS (*n* = 7, 18%). In these specialties, the goal is not only addressing patient concerns in the chair but attempting to predict outcomes and trajectories of treating interventions. In the field of orthodontics, AI can be utilized to predict the need for orthodontic interventions through the analysis of imaging techniques such as lateral cephalograms. Furthermore, predictive AI in orthodontics has been utilized to assess the need for orthognathic surgery solely based on facial photographs.

In terms of imaging, OPGs (*n* = 90, 33%) and intra‐oral radiographs (*n* = 56, 20%) were the most common. Clinicians are well‐versed in acquiring and interpreting OPGs as they are commonly performed for patients and are readily available. OPGs are clinically relevant to many different specialties such as OMFS and periodontics, making them a useful imaging modality for AI algorithms. In contrast, intra‐oral radiographs (bitewings and periapicals) are imperative for caries detection and diagnosis, which is the reason why over 19% of articles used this modality for their algorithm.

The majority of studies used neural networks to generate AI models. The most frequently reported type of neural network was CNN (*n* = 204, 74%) that mimics the visual processing capabilities of the human brain, hence the name. These models excel at extracting features from images via hierarchical feature extraction and are capable of learning spatial invariance to recognize patterns and objects regardless of position or orientation within the image (Alzubaidi et al. 2021). This makes it a superior tool for dentistry where there can be a lot of variability in the imaging of patients.

### 4.2 Research Gaps

The use of AI in dentistry is still an emerging field, with limited application to clinical practice at this point. Some barriers include the need for large volumes of training data, inconsistency in data annotation, and population demographics. Although some studies had ample sample sizes in the tens of thousands, others were well below a hundred, which can impact the training and resulting performance of the algorithms. Although AI models are meant to learn independently from a data set, initially, they may require varying extents of annotation within subsets and training. Due to the requirement of large quantities of training data, there can be multiple annotators, introducing variability in identifying the necessary features for the algorithm to use. Another limitation in this scoping review and applicability to clinical practice in Australia is the lack of representation in population demographics—the majority of the studies were from mainly Asian countries such as South Korea and China. Although dental practice may be similar in these regions, the epidemiology and data used for training may not apply as closely to populations in Australia.

### 4.3 Implications and Future Applications

Clinical decision‐making in dentistry is a complex process as numerous aspects of dental examination rely on indirect measures. Therefore, dental clinicians are required to have profound knowledge with respect to basic principles and appropriate evaluation regarding diagnostic tests and published literature. Nevertheless, discrepancies often arise among experienced practitioners regarding clinical decision‐making. In the field of dentistry, a gold standard is defined as the benchmark with known results that is the best available under reasonable conditions (Cardoso et al. 2014). Introducing benchmarks through AI can be beneficial in facilitating dental practitioners, especially less experienced ones, to make more informed clinical decisions.

A diverse image modality can be processed by AI, which CT and MRI require knowledge of systemic medicine and can be a challenge for dental practitioners. AI‐assisted decision‐making has the potential to benefit dentists in making more informed diagnoses. However, there is currently no standardization with respect to the amount of data set to be trained per algorithm as well as demographics mentioned in the previous section, thus potentially challenging the accuracy and validity of clinical AI applications.

Segmentation plays a crucial role in processing data from imagery and has been applied widely including for the development of computer‐assisted diagnosis (CAD) using various different modalities of radiological images, such as cephalograms, OPGs and CTs. The mechanism involves processing clusters of images into coherent sub‐regions based on extracted features, for instance, texture attributes (Seo et al. 2020). Forty‐five articles evaluating AI segmentation on different imagery modalities in dentistry were included in this review. Although the technology is not directly contributing to decision‐making, the papers investigating segmentation showed great proof‐of‐concept potential and preliminary work toward processes ranging from automatic landmark identification to tooth segmentation without manual labeling.

AI's potential in dentistry has the ability to extend beyond AI‐assisted diagnosis during appointments. For example, six papers revealed novel AI‐driven detection of oral lesions at home or in remote settings. They are excluded from this review as the application did not directly involve in‐chair practice. However, it is important to encourage research in these areas as it could improve early detection, prevent diagnostic delays, and encourage patient–clinician communication. Another study by Schwendicke et al. (2022) evaluated the cost‐effectiveness of AI diagnosis. Although there is currently no formal significant evidence proving that AI diagnosis is more cost‐effective, it is postulated that AI applications can be advantageous in reducing practitioner workload and chairside time. These factors may benefit both clinicians and patients as overtreatment and misdiagnosis are minimized, and resources become better allocated.

Despite AI‐assisted decision‐making showing great potential, it is important to discuss the ethical issues involved. AI training and application require the input of patient data, meaning there should be regulations introduced in relation to privacy and data security. The use of patient data as part of AI training suggests that patient information will continuously be applied as part of the algorithm; hence, patients should be informed appropriately. Human oversight and associated liability and accountability can be a challenge in AI‐assisted decision‐making as a large portion of the articles reviewed were designed to assist less‐experienced practitioners. This raises questions such as whether a less‐experienced clinician is able to correct the error made by AI, and if AI concludes an incorrect diagnosis, who will be held liable for the potential damages to the patient.

## 5 Conclusions

This review demonstrated the diverse use of AI in dentistry, especially in the past 5 years. AI is progressively gaining a foothold in clinical dentistry and is used for a wide array of purposes across all fields of dentistry. Current studies adopted a variety of machine learning and deep learning techniques for analysis across several dental image modalities to assist in clinical decision‐making and in efforts toward achieving better patient outcomes. Most studies provided initial proof of concept and advocated for both further development and future implementation of AI in dentistry. Therefore, systematic reviews and meta‐analyses of the current literature should be performed to critically appraise the quality and validity of these studies to objectively evaluate the reliability of AI use in dental practice. In addition, future studies in clinical settings across different countries and the standardization of training protocol objectives should be conducted to fill in existing research gaps.

## Author Contributions

*Investigation, formal analysis, data curation, writing–original draft*: Wei Chen, Monisha Dhawan, Jonathan Liu, Damie Ing, Kruti Mehta, and Daniel Tran. *Conceptualization, writing–review and editing, supervision*: Daniel Lawrence and Max Ganhewa. *Conceptualization, methodology, writing–review and editing, supervision, project administration*: Nicola Cirillo.

## Ethics Statement

This is a review article and did not require ethical clearance.

## Conflicts of Interest

M.G. is the founder and CEO of CoTreat Pty Ltd, Melbourne, Victoria, Australia.

## Supporting information

Supporting information.

## Data Availability

The data that support the findings of this study are available in the Supporting Information of this article.
