# Supplementary material for: Mapping the Use of Artificial Intelligence–Based Image Analysis for Clinical Decision‐Making in Dentistry: A Scoping Review
Source: Clin Exp Dent Res. 2024 Nov 26;10(6):e70035. doi: 10.1002/cre2.70035 (PMC11599430; doi:10.1002/cre2.70035)
Supplement: Supplementary file 1 — Supporting information. [file CRE2-10-e70035-s001.docx]

**Supplementary Material**


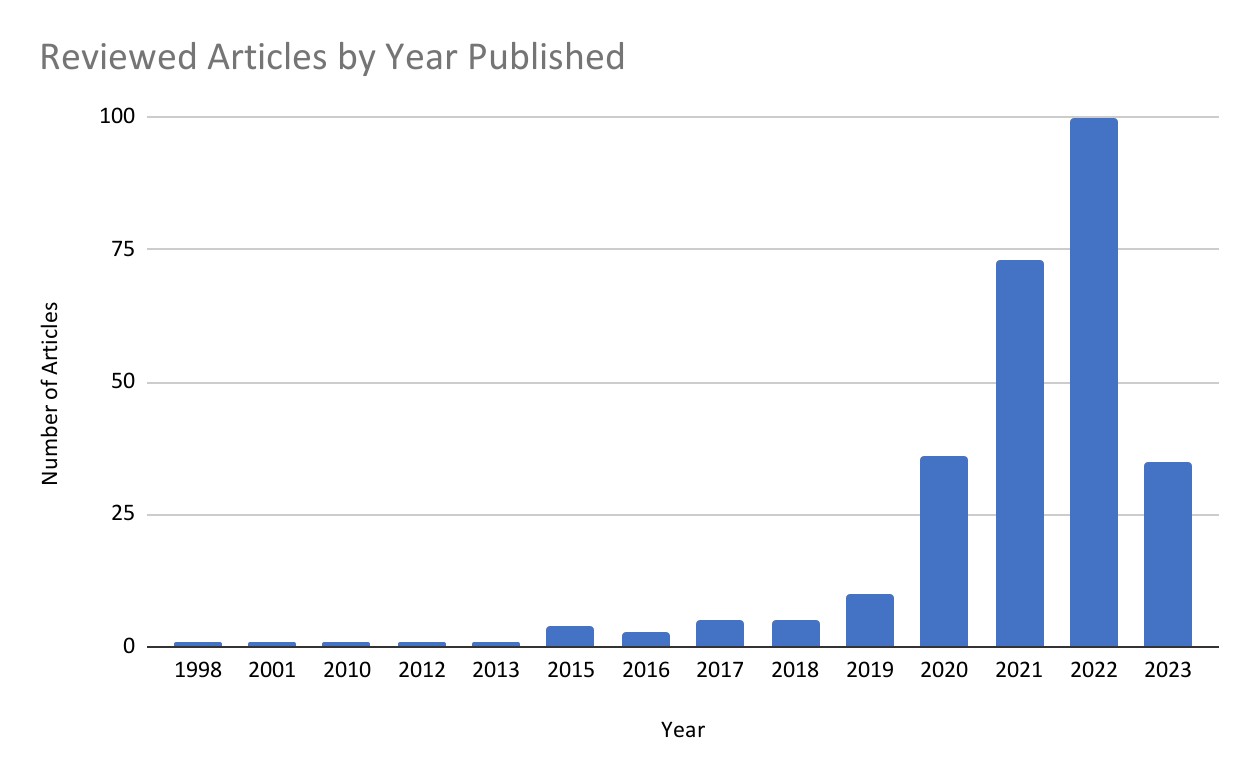


**Supplementary Figure 1.** Articles included in this scoping review by year of publication.


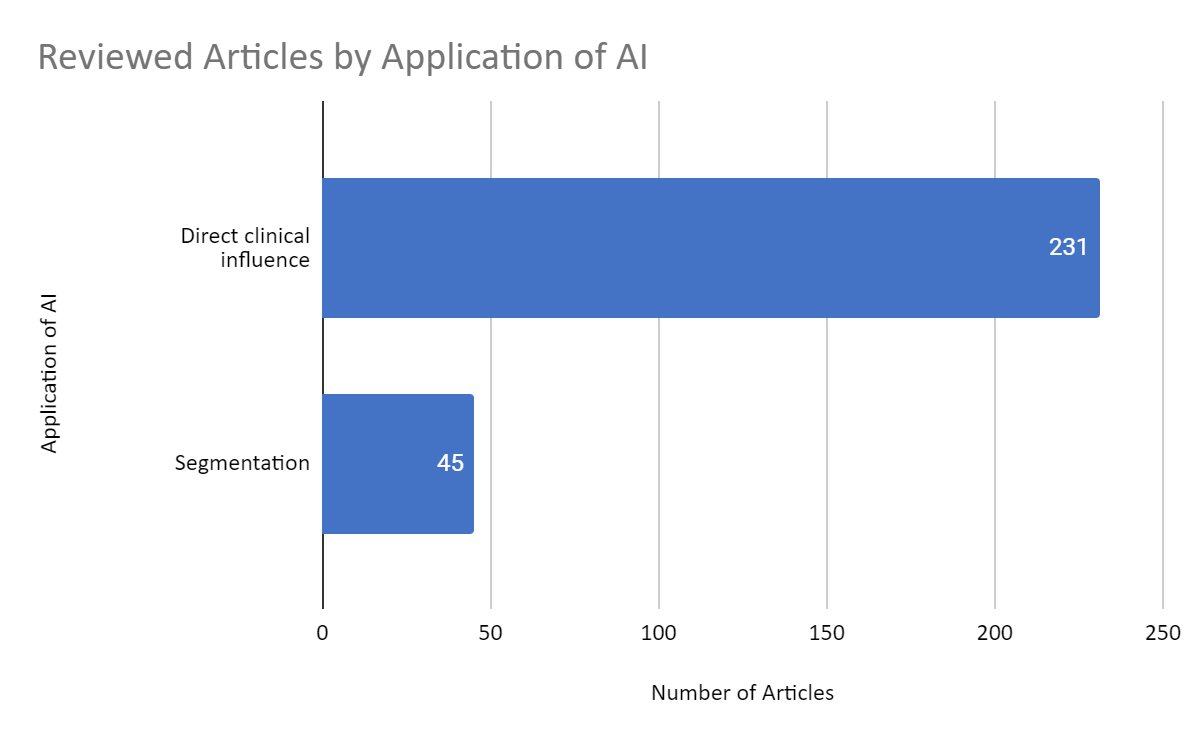


**Supplementary Figure 2:** The distribution of reviewed articles by application of AI.


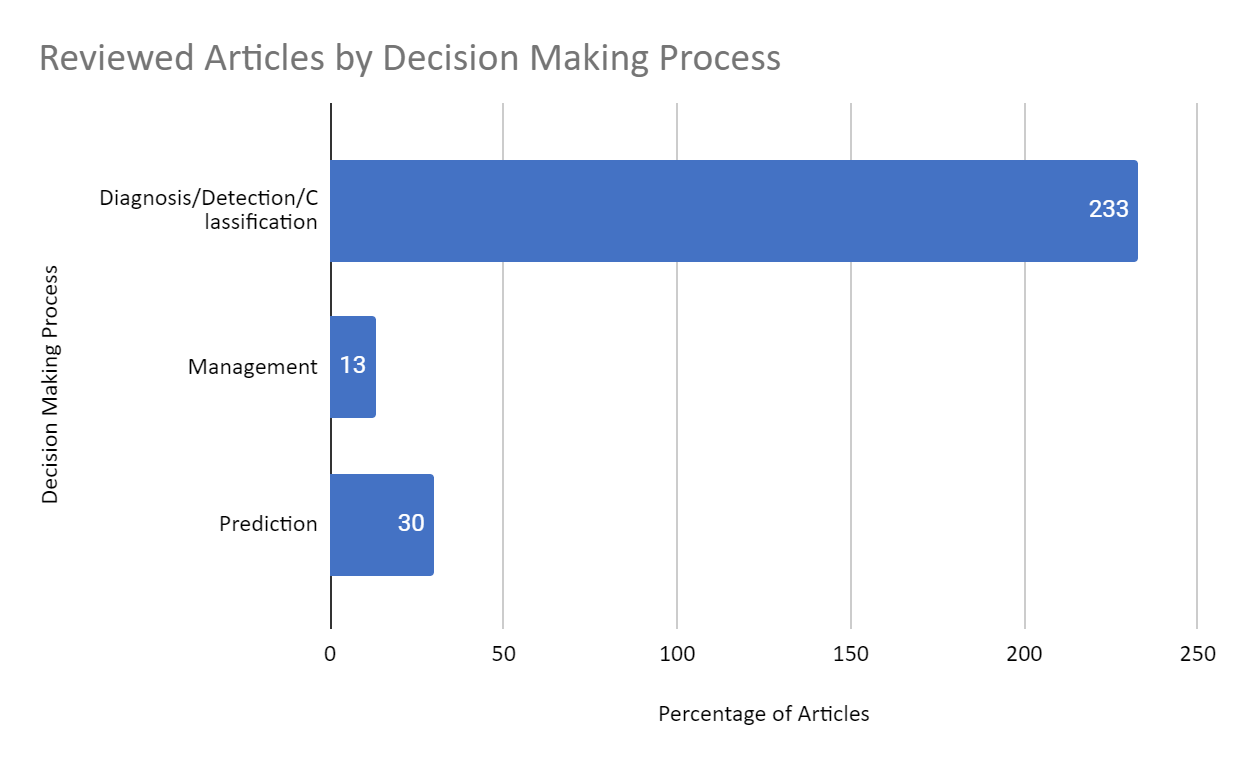


**Supplementary Figure 3:** AI-assisted decision-making process involved in the reviewed articles.


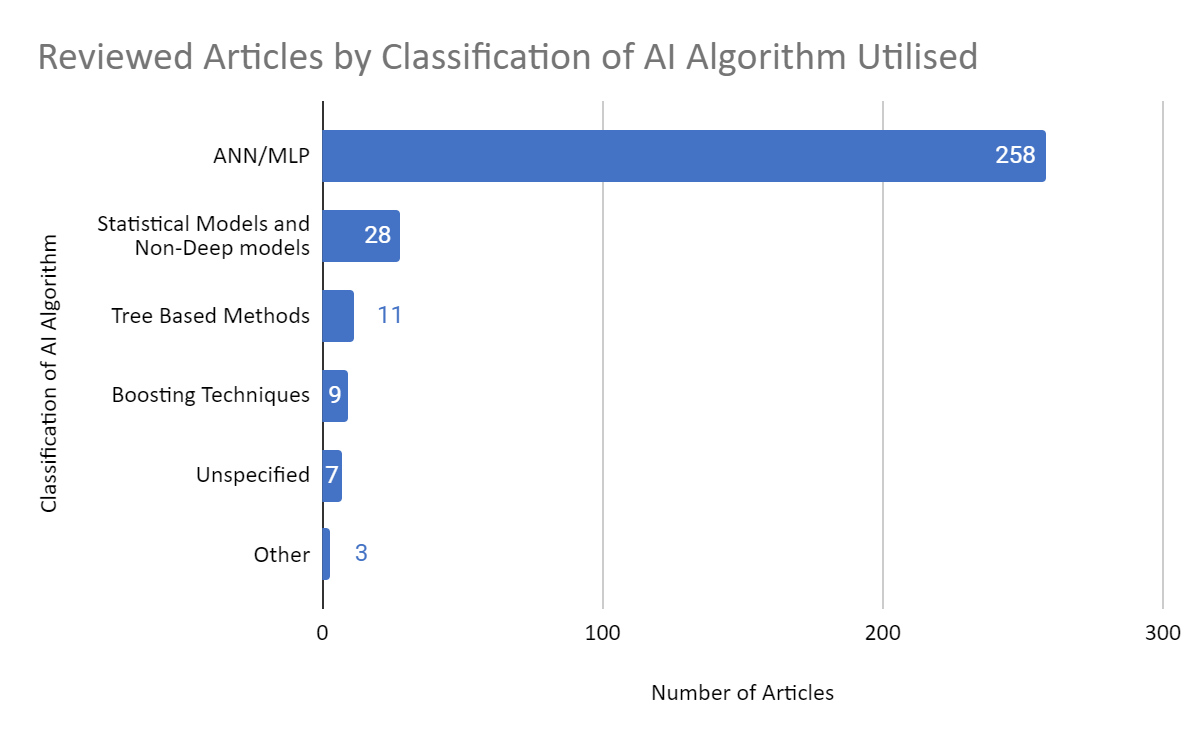


**Supplementary Figure 4:** The distribution of reviewed articles by the classification of AI algorithms used. ANN (artificial neural network), MLP (multilayer preceptron)


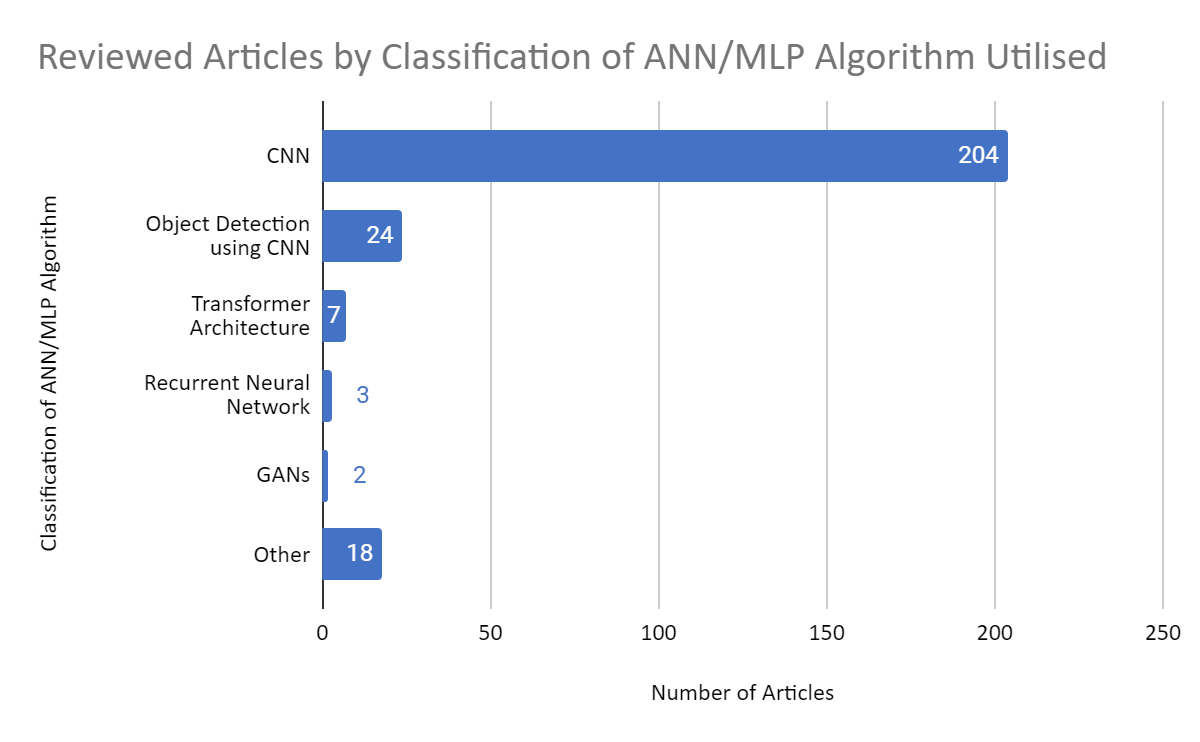


**Supplementary Figure 5:** The distribution of reviewed articles by the classification of ANN (artificial neural network)/MLP (multilayer preceptron) algorithms used. CNN (convolutional neural network) GAN (generative adversarial network)


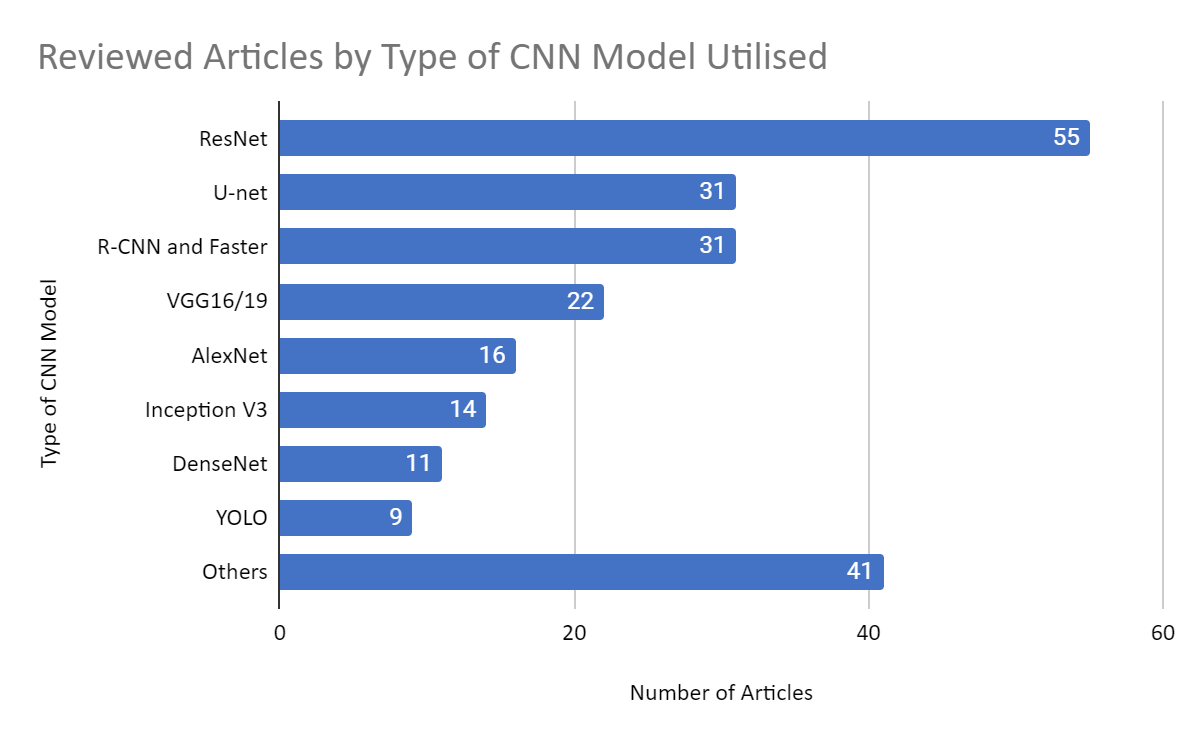


**Supplementary Figure 6:** The distribution of reviewed articles by the classification of CNN (Convolutional Neural Network) algorithms used. ResNet (residual neural network) R-CNN (region-based convolutional neural network) YOLO (you only look once)

**Supplementary Table 1:** PRISMA for Scoping Reviews (PRISMA-ScR) Checklist

| **SECTION** | **ITEM** | **PRISMA-ScR CHECKLIST ITEM** | **REPORTED ON PAGE #** |
| --- | --- | --- | --- |
| **TITLE** | | | |
| Title | 1 | Identify the report as a scoping review. | 1 |
| **ABSTRACT** | | | |
| Structured summary | 2 | Provide a structured summary that includes (as applicable): background, objectives, eligibility criteria, sources of evidence, charting methods, results, and conclusions that relate to the review questions and objectives. | 2 |
| **INTRODUCTION** | | | |
| Rationale | 3 | Describe the rationale for the review in the context of what is already known. Explain why the review questions/objectives lend themselves to a scoping review approach. | 3 |
| Objectives | 4 | Provide an explicit statement of the questions and objectives being addressed with reference to their key elements (e.g., population or participants, concepts, and context) or other relevant key elements used to conceptualize the review questions and/or objectives. | 4 |
| **METHODS** | | | |
| Protocol and registration | 5 | Indicate whether a review protocol exists; state if and where it can be accessed (e.g., a Web address); and if available, provide registration information, including the registration number. | 4 |
| Eligibility criteria | 6 | Specify characteristics of the sources of evidence used as eligibility criteria (e.g., years considered, language, and publication status), and provide a rationale. | 5-6, 60 |
| Information sources* | 7 | Describe all information sources in the search (e.g., databases with dates of coverage and contact with authors to identify additional sources), as well as the date the most recent search was executed. | 4-5 |
| Search | 8 | Present the full electronic search strategy for at least 1 database, including any limits used, such that it could be repeated. | 4 |
| Selection of sources of evidence† | 9 | State the process for selecting sources of evidence (i.e., screening and eligibility) included in the scoping review. | 5-6 |
| Data charting process‡ | 10 | Describe the methods of charting data from the included sources of evidence (e.g., calibrated forms or forms that have been tested by the team before their use, and whether data charting was done independently or in duplicate) and any processes for obtaining and confirming data from investigators. | 6 |
| Data items | 11 | List and define all variables for which data were sought and any assumptions and simplifications made. | 6 |
| Critical appraisal of individual sources of evidence§ | 12 | If done, provide a rationale for conducting a critical appraisal of included sources of evidence; describe the methods used and how this information was used in any data synthesis (if appropriate). | NO |
| Synthesis of results | 13 | Describe the methods of handling and summarizing the data that were charted. | 6-7 |
| **RESULTS** | | | |
| Selection of sources of evidence | 14 | Give numbers of sources of evidence screened, assessed for eligibility, and included in the review, with reasons for exclusions at each stage, ideally using a flow diagram. | 7 |
| Characteristics of sources of evidence | 15 | For each source of evidence, present characteristics for which data were charted and provide the citations. | 7 |
| Critical appraisal within sources of evidence | 16 | If done, present data on critical appraisal of included sources of evidence (see item 12). | NO |
| Results of individual sources of evidence | 17 | For each included source of evidence, present the relevant data that were charted that relate to the review questions and objectives. | 7-10 |
| Synthesis of results | 18 | Summarize and/or present the charting results as they relate to the review questions and objectives. | 7-10, 54-59 |
| **DISCUSSION** | | | |
| Summary of evidence | 19 | Summarize the main results (including an overview of concepts, themes, and types of evidence available), link to the review questions and objectives, and consider the relevance to key groups. | 11-12 |
| Limitations | 20 | Discuss the limitations of the scoping review process. | 12-13 |
| Conclusions | 21 | Provide a general interpretation of the results with respect to the review questions and objectives, as well as potential implications and/or next steps. | 15 |
| **FUNDING** | | | |
| Funding | 22 | Describe sources of funding for the included sources of evidence, as well as sources of funding for the scoping review. Describe the role of the funders of the scoping review. | 15 |

*From:* Tricco AC, Lillie E, Zarin W, O'Brien KK, Colquhoun H, Levac D, et al. PRISMA Extension for Scoping Reviews (PRISMAScR): Checklist and Explanation. Ann Intern Med. 2018;169:467–473. [doi: 10.7326/M18-0850](http://annals.org/aim/fullarticle/2700389/prisma-extension-scoping-reviews-prisma-scr-checklist-explanation).

**Supplementary Table 2:** Inclusion and exclusion criteria used for this scoping review.

| **Inclusion Criteria** | **Exclusion Criteria** |
| --- | --- |
| Diagnosis/Detection/Classification | No imaging input |
| Management | No artificial intelligence |
| Prediction | In vitro/ex vivo/extracted teeth |
| All fields of clinical dentistry | Not peer-reviewed |
| All medical and dental imaging modalities | Review |
| All types of artificial intelligence algorithms | Could not retrieve full text |
| AI utilised for segmentation | Not relevant to ‘clinical’ decision making |
| AI with direct clinical influence | Uncommon imaging |
| Primary evidence | Forensic Dentistry |
|  | Traditional Chinese Medicine |
|  | Histology |
|  | Tele-dentistry |

**Supplementary Table 3:** Summary of relevant data from included articles

| **References** | **Part of Decision Making** | **Field of Dentistry** | **Application of AI** | **AI Classification** | **Imaging Modality** | **Size of Dataset** |
| --- | --- | --- | --- | --- | --- | --- |
| (Agarwal et al., 2022) | Diagnosis/Detection/Classification | Oral Medicine | Direct clinical influence | CNN | CT | 1755 |
| (Ahn et al., 2021) | Diagnosis/Detection/Classification | Paediatric Dentistry | Direct clinical influence | CNN | Intra-oral Radiograph | 1100 |
| (Alalharith et al., 2020) | Diagnosis/Detection/Classification | Periodontics | Direct clinical influence | Object Detection using CNN | Clinical photograph | 134 |
| (Al-Ghamdi et al., 2022) | Diagnosis/Detection/Classification | Cariology, OMFS | Direct clinical influence | CNN | OPG | 116 |
| (Aljabri et al., 2022) | Diagnosis/Detection/Classification | Orthodontics | Direct clinical influence | CNN | Intra-oral Radiograph | 416 |
| (Al-Ma'aitah & AlZubi, 2018) | Diagnosis/Detection/Classification | Oral Medicine | Direct clinical influence | Recurrent Neural Network | Intra-oral Radiograph |  |
| (Alotaibi et al., 2022) | Diagnosis/Detection/Classification | Periodontics | Direct clinical influence | CNN | Intra-oral Radiograph | 1724 |
| (Andrade et al., 2023) | Diagnosis/Detection/Classification | Cariology, Periodontics, Paediatric Dentistry | Direct clinical influence | CNN | Clinical photograph | 576 |
| (Ariji et al., 2019) | Diagnosis/Detection/Classification | Oral Medicine | Direct clinical influence | CNN | CT | 45 |
| (Ariji et al., 2021) | Diagnosis/Detection/Classification | Oral Medicine | Direct clinical influence | CNN | CT | 365 |
| (Ariji et al., 2020) | Diagnosis/Detection/Classification | Oral Medicine | Direct clinical influence | CNN | CT | 703 |
| (Askar et al., 2021) | Diagnosis/Detection/Classification | Cariology, Paediatric Dentistry | Direct clinical influence | CNN | Clinical photograph | 434 |
| (Awais et al., 2020) | Diagnosis/Detection/Classification | Oral Medicine | Direct clinical influence | Statistical Models and Non-Deep models | Clinical photograph | 22 |
| (Ayidh Alqahtani et al., 2023) | Diagnosis/Detection/Classification | Orthodontics | Direct clinical influence | CNN | CBCT | 215 |
| (Banar et al., 2020) | Diagnosis/Detection/Classification | Dental Radiography, OMFS | Direct clinical influence | CNN | OPG | 400 |
| (Bayraktar & Ayan, 2022) | Diagnosis/Detection/Classification | Cariology | Direct clinical influence | CNN | Intra-oral Radiograph | 1000 |
| (Berdouses et al., 2015) | Diagnosis/Detection/Classification | Cariology | Direct clinical influence | Tree Based Methods | Clinical photograph | 103 |
| (Bharathi et al., 2022) | Diagnosis/Detection/Classification | Systemic Medicine | Direct clinical influence | CNN, Statistical Models and Non-Deep models | Clinical photograph | 500 |
| (Bhattacharjee, 2022) | Diagnosis/Detection/Classification | Cariology | Direct clinical influence | CNN | Clinical photograph | 314 |
| (Bispo et al., 2021) | Diagnosis/Detection/Classification | OMFS | Direct clinical influence | CNN | CT | 2500 |
| (Buyuk et al., 2022) | Management | OMFS | Direct clinical influence | CNN | OPG | 1880 |
| (Buyuk et al., 2023) | Diagnosis/Detection/Classification | Endodontics | Direct clinical influence | Recurrent Neural Network, CNN | OPG | 915 |
| (Camalan et al., 2021) | Diagnosis/Detection/Classification | Oral Medicine | Direct clinical influence | CNN | Clinical photograph | 54 |
| (Cantu et al., 2020) | Diagnosis/Detection/Classification | Cariology | Direct clinical influence | CNN | Intra-oral Radiograph | 3686 |
| (Carmody et al., 2001) | Diagnosis/Detection/Classification | Unspecified | Segmentation | Unspecified | Intra-oral Radiograph | 32 |
| (Casalegno et al., 2019) | Diagnosis/Detection/Classification | Cariology | Direct clinical influence | CNN | Clinical photograph | 217 |
| (Celik, 2022) | Diagnosis/Detection/Classification | OMFS | Direct clinical influence | Object Detection using CNN | OPG | 440 |
| (Chai et al., 2022) | Diagnosis/Detection/Classification | Oral Medicine | Direct clinical influence | CNN | CBCT | 350 |
| (Chandrashekar et al., 2022) | Diagnosis/Detection/Classification | Unspecified | Segmentation | CNN | Intra-oral Radiograph | 1500 |
| (Chang et al., 2020) | Diagnosis/Detection/Classification | Periodontics | Direct clinical influence | CNN | OPG | 518 |
| (Chang et al., 2022) | Diagnosis/Detection/Classification | Periodontics | Direct clinical influence | CNN | Intra-oral Radiograph | 2362 |
| (H. Chen et al., 2021) | Diagnosis/Detection/Classification | Cariology, Endodontics, Periodontics | Direct clinical influence | CNN | Intra-oral Radiograph | 2900 |
| (Q. Chen et al., 2021) | Diagnosis/Detection/Classification | Unspecified | Segmentation | CNN | 3D dental scanner | 2052 |
| (Chen et al., 2023) | Diagnosis/Detection/Classification | Cariology | Direct clinical influence | CNN, Object Detection using CNN | Intra-oral Radiograph | 978 |
| (Cheng & Wang, 2019) | Diagnosis/Detection/Classification | OMFS | Segmentation | Statistical Models and Non-Deep models | Intra-oral Radiograph | 500 |
| (Chifor et al., 2022) | Diagnosis/Detection/Classification | Periodontics | Direct clinical influence | CNN, Object Detection using CNN | Ultrasound | 2135 |
| (Choi et al., 2021) | Diagnosis/Detection/Classification | OMFS | Direct clinical influence | Object Detection using CNN, Statistical Models and Non-Deep models | OPG | 1189 |
| (Choi et al., 2022) | Management | OMFS | Direct clinical influence | CNN | OPG | 571 |
| (Chung et al., 2021) | Diagnosis/Detection/Classification | Unspecified | Segmentation | CNN, Object Detection using CNN | OPG | 818 |
| (Chung et al., 2020) | Diagnosis/Detection/Classification | Orthodontics | Segmentation | CNN | CBCT | 175 |
| (Chung et al., 2022) | Diagnosis/Detection/Classification | Orthodontics | Direct clinical influence | Unspecified | CBCT, Lateral Cephalogram | 30 |
| (Cieślińska et al., 2022) | Diagnosis/Detection/Classification | Paediatric Dentistry | Direct clinical influence | ANN/MLP | OPG | 300 |
| (Cui et al., 2022) | Diagnosis/Detection/Classification | Unspecified | Segmentation | Unspecified | CBCT | 4938 |
| (Cui et al., 2021) | Diagnosis/Detection/Classification | Orthodontics | Segmentation | ANN/MLP | 3D dental scanner | 2000 |
| (Danks et al., 2021) | Diagnosis/Detection/Classification | Periodontics | Direct clinical influence | CNN | Intra-oral Radiograph | 340 |
| (Dayı et al., 2023) | Diagnosis/Detection/Classification | Cariology | Direct clinical influence | CNN | OPG | 504 |
| (De Angelis et al., 2022) | Diagnosis/Detection/Classification | Unspecified | Segmentation | Unspecified | OPG | 120 |
| (De Araujo Faria et al., 2021) | Prediction | Cariology | Direct clinical influence | ANN/MLP | OPG | 15 |
| (De Tobel et al., 2017) | Prediction | Paediatric Dentistry, Orthodontics | Direct clinical influence | CNN | OPG | 400 |
| (Ding et al., 2021) | Diagnosis/Detection/Classification | OMFS | Direct clinical influence | CNN | Clinical photograph | 912 |
| (Dong et al., 2018) | Management | Oral Medicine | Direct clinical influence | Statistical Models and Non-Deep models | Digital Infrared Thermal Imaging | 90 |
| (Dot et al., 2022) | Diagnosis/Detection/Classification | Orthodontics | Direct clinical influence | CNN | CT | 198 |
| (Du et al., 2018) | Diagnosis/Detection/Classification | Dental Radiography | Segmentation | CNN | OPG | 5166 |
| (Dubuc et al., 2022) | Diagnosis/Detection/Classification | Oral Medicine | Direct clinical influence | ANN/MLP, Other, Boosting Techniques, Tree Based Methods, Statistical Models and Non-Deep models | Clinical photograph | 299 |
| (Duman et al., 2023) | Diagnosis/Detection/Classification | Paediatric Dentistry | Direct clinical influence | CNN | OPG | 434 |
| (Edvardsen et al., 2022) | Diagnosis/Detection/Classification | Orthodontics, OMFS | Direct clinical influence | CNN | Intra-oral Radiograph | 5197 |
| (Endres et al., 2020) | Diagnosis/Detection/Classification | Endodontics | Direct clinical influence | CNN | OPG | 2902 |
| (Estai et al., 2022) | Diagnosis/Detection/Classification | Cariology | Direct clinical influence | CNN | Intra-oral Radiograph | 2468 |
| (Etemad et al., 2021) | Management | Orthodontics | Direct clinical influence | Tree Based Methods | Lateral Cephalogram | 838 |
| (Ezhov et al., 2021) | Diagnosis/Detection/Classification | Oral Medicine, Orthodontics, Dental Radiography | Direct clinical influence | CNN | CBCT | 1246 |
| (Figueroa et al., 2022) | Diagnosis/Detection/Classification | Oral Medicine | Direct clinical influence | CNN | Clinical photograph | 1200 |
| (Firestone et al., 1998) | Diagnosis/Detection/Classification | Cariology | Direct clinical influence | Unspecified | Intra-oral Radiograph | 102 |
| (Flügge et al., 2023) | Diagnosis/Detection/Classification | Oral Medicine | Direct clinical influence | Transformer Architecture | Clinical photograph | 1406 |
| (Fu et al., 2020) | Diagnosis/Detection/Classification | Oral Medicine | Direct clinical influence | CNN | Clinical photograph | 44409 |
| (Fujima et al., 2020) | Prediction | Oral Medicine | Direct clinical influence | CNN | CT | 83 |
| (Fujima et al., 2021) | Prediction | Oral Medicine | Direct clinical influence | Statistical Models and Non-Deep models | CT | 99 |
| (Fukuda et al., 2020) | Diagnosis/Detection/Classification | Endodontics | Direct clinical influence | CNN | OPG | 300 |
| (Görürgöz et al., 2022) | Diagnosis/Detection/Classification | Unspecified | Segmentation | Object Detection using CNN | Intra-oral Radiograph | 1686 |
| (Galibourg et al., 2021) | Prediction | Paediatric Dentistry, Orthodontics | Direct clinical influence | Boosting Techniques, Statistical Models and Non-Deep models, Tree Based Methods, ANN/MLP | Clinical photograph, OPG | 3605 |
| (Gao et al., 2022) | Diagnosis/Detection/Classification | Dental Radiography | Direct clinical influence | CNN, Transformer Architecture | CBCT | 25 |
| (García-Cañas et al., 2023) | Diagnosis/Detection/Classification | Cariology | Direct clinical influence | CNN, Object Detection using CNN | Intra-oral Radiograph | 300 |
| (Geetha et al., 2020) | Diagnosis/Detection/Classification | Cariology | Direct clinical influence | CNN | Intra-oral Radiograph | 105 |
| (Gerhardt et al., 2022) | Diagnosis/Detection/Classification | Paediatric Dentistry, Orthodontics, Prosthodontics | Direct clinical influence | CNN | CBCT | 175 |
| (Guo et al., 2022) | Diagnosis/Detection/Classification | Cariology, Endodontics | Direct clinical influence | CNN | Clinical photograph | 800 |
| (Hamdan et al., 2022) | Diagnosis/Detection/Classification | Endodontics | Direct clinical influence | CNN | Intra-oral Radiograph | 68 |
| (Han et al., 2022) | Prediction | Orthodontics, Paediatric Dentistry | Direct clinical influence | CNN | OPG | 10257 |
| (Hao et al., 2022) | Diagnosis/Detection/Classification | Orthodontics | Segmentation | CNN, Statistical Models and Non-Deep models | 3D dental scanner | 4000 |
| (Hernandez et al., 2021) | Diagnosis/Detection/Classification | Dental Radiography | Direct clinical influence | CNN | CT | 83676 |
| (Hiraiwa et al., 2019) | Diagnosis/Detection/Classification | Endodontics | Direct clinical influence | CNN | OPG | 760 |
| (Hosntalab et al., 2010) | Diagnosis/Detection/Classification | Unspecified | Segmentation | ANN/MLP | CT | 30 |
| (Hou et al., 2023) | Diagnosis/Detection/Classification | Dental Radiography | Segmentation | CNN | OPG | 1500 |
| (Hu et al., 2022) | Diagnosis/Detection/Classification | Endodontics | Direct clinical influence | CNN | CBCT | 276 |
| (Huang et al., 2022) | Prediction | OMFS | Direct clinical influence | CNN, Statistical Models and Non-Deep models | CBCT | 12624 |
| (Huang et al., 2021) | Diagnosis/Detection/Classification | Oral Medicine | Direct clinical influence | CNN | CBCT | 353 |
| (Hur et al., 2021) | Prediction | Cariology | Direct clinical influence | ANN/MLP, Statistical Models and Non-Deep models, Tree Based Methods | OPG, CBCT | 1321 |
| (Hwang et al., 2017) | Diagnosis/Detection/Classification | Systemic Medicine | Direct clinical influence | Statistical Models and Non-Deep models | OPG | 454 |
| (Ishibashi et al., 2022) | Diagnosis/Detection/Classification | Oral Medicine | Direct clinical influence | Unspecified | OPG | 224 |
| (Issa et al., 2023) | Diagnosis/Detection/Classification | Endodontics | Direct clinical influence | CNN | Intra-oral Radiograph | 20 |
| (Issa et al., 2022) | Diagnosis/Detection/Classification | Oral Medicine | Direct clinical influence | Boosting Techniques | Clinical photograph | 80 |
| (James et al., 2021) | Diagnosis/Detection/Classification | Oral Medicine | Direct clinical influence | ANN/MLP, Statistical Models and Non-Deep models | OCT | 232 |
| (Jang et al., 2022) | Diagnosis/Detection/Classification | OMFS | Direct clinical influence | Object Detection using CNN | Intra-oral Radiograph | 300 |
| (Jeon & Lee, 2021) | Diagnosis/Detection/Classification | Orthodontics | Direct clinical influence | CNN | CBCT | 35 |
| (Jeon et al., 2021) | Diagnosis/Detection/Classification | Endodontics | Direct clinical influence | CNN | OPG, CBCT | 2040 |
| (Jeong et al., 2022) | Prediction | OMFS | Direct clinical influence | ANN/MLP | CT | 269 |
| (Jeong et al., 2020) | Diagnosis/Detection/Classification | Orthodontics | Direct clinical influence | CNN | Clinical photograph | 822 |
| (Jiang et al., 2023) | Diagnosis/Detection/Classification | Orthodontics | Segmentation | CNN | Lateral Cephalogram | 9870 |
| (Jiang et al., 2021) | Diagnosis/Detection/Classification | Cariology | Direct clinical influence | Transformer Architecture | Clinical photograph | 8554 |
| (Jiang et al., 2022) | Diagnosis/Detection/Classification | Periodontics | Direct clinical influence | Object Detection using CNN | OPG | 640 |
| (Jones et al., 2022) | Diagnosis/Detection/Classification | Cariology | Direct clinical influence | CNN | Clinical photograph | 130 |
| (Joo et al., 2023) | Prediction | Orthodontics | Direct clinical influence | CNN | OPG | 910 |
| (Jubair et al., 2022) | Diagnosis/Detection/Classification | Oral Medicine | Direct clinical influence | CNN | Phone Images | 716 |
| (Jung et al., 2023) | Diagnosis/Detection/Classification | OMFS | Direct clinical influence | CNN | OPG | 858 |
| (Jurczyszyn et al., 2020) | Diagnosis/Detection/Classification | Oral Medicine | Direct clinical influence | ANN/MLP | Clinical photograph | 35 |
| (Kühnisch et al., 2022) | Diagnosis/Detection/Classification | Cariology | Direct clinical influence | CNN | Clinical photograph | 2417 |
| (Kabir et al., 2022) | Diagnosis/Detection/Classification | Cariology, Periodontics | Segmentation | CNN | Intra-oral Radiograph, OPG | 2038 |
| (Kats et al., 2021) | Diagnosis/Detection/Classification | Dental Radiography | Direct clinical influence | CNN | Lateral Cephalogram, OPG | 620 |
| (Kavitha et al., 2015) | Diagnosis/Detection/Classification | Systemic Medicine | Direct clinical influence | Statistical Models and Non-Deep models | OPG | 141 |
| (Kavitha et al., 2012) | Diagnosis/Detection/Classification | Systemic Medicine | Direct clinical influence | Statistical Models and Non-Deep models | OPG | 100 |
| (Kearney et al., 2022) | Prediction | Periodontics | Direct clinical influence | GANs | Intra-oral Radiograph | 80326 |
| (Kempers et al., 2023) | Diagnosis/Detection/Classification | OMFS | Direct clinical influence | CNN | OPG | 863 |
| (Kim, D. et al., 2021) | Prediction | Orthodontics, Paediatric Dentistry | Direct clinical influence | Statistical Models and Non-Deep models | Lateral Cephalogram, Hand-wrist Xray | 499 |
| (Kim, J. et al., 2021) | Diagnosis/Detection/Classification | Orthodontics | Segmentation | CNN | Lateral Cephalogram | 3150 |
| (Kim, H. et al., 2023) | Prediction | Orthodontics, Paediatric Dentistry | Direct clinical influence | Transformer Architecture | Hand-wrist Xray | 2593 |
| (Kim, J. et al., 2022) | Diagnosis/Detection/Classification | Paediatric Dentistry | Direct clinical influence | CNN | OPG | 988 |
| (Kim, J. et al., 2020) | Diagnosis/Detection/Classification | OMFS | Direct clinical influence | CNN | Intra-oral Radiograph | 801 |
| (Kim, S. et al., 2021) | Prediction | Paediatric Dentistry, Orthodontics | Direct clinical influence | CNN | OPG | 1586 |
| (Kim, Y. H. et al., 2021) | Diagnosis/Detection/Classification | OMFS, Periodontics, Orthodontics | Segmentation | CNN | CBCT | 12800 |
| (Kise et al., 2019) | Diagnosis/Detection/Classification | Oral Medicine | Direct clinical influence | CNN | CT | 500 |
| (Kök et al., 2021) | Prediction | Orthodontics, Paediatric Dentistry | Direct clinical influence | ANN/MLP | Lateral Cephalogram, Hand-wrist Xray | 419 |
| (Kositbowornchai et al., 2013) | Diagnosis/Detection/Classification | Endodontics | Direct clinical influence | ANN/MLP | Intra-oral Radiograph | 200 |
| (Krois et al., 2019) | Diagnosis/Detection/Classification | Periodontics | Direct clinical influence | CNN | OPG | 2001 |
| (Krois et al., 2021) | Diagnosis/Detection/Classification | Unspecified | Segmentation | CNN | OPG | 5008 |
| (Kubo et al., 2022) | Prediction | Oral Medicine | Direct clinical influence | Tree Based Methods, Statistical Models and Non-Deep models, Boosting Techniques | CT | 161 |
| (Kunz et al., 2020) | Diagnosis/Detection/Classification | Orthodontics | Direct clinical influence | CNN | Lateral Cephalogram | 1792 |
| (Kuwada et al., 2021) | Diagnosis/Detection/Classification | OMFS | Direct clinical influence | CNN | CT | 383 |
| (Kuwada et al., 2020) | Diagnosis/Detection/Classification | Paediatric Dentistry | Direct clinical influence | CNN | OPG | 550 |
| (Kwak et al., 2020) | Diagnosis/Detection/Classification | Oral Medicine, OMFS | Direct clinical influence | CNN | CBCT | 9818 |
| (Kwon et al., 2020) | Diagnosis/Detection/Classification | Oral Medicine | Direct clinical influence | CNN | Intra-oral Radiograph | 1282 |
| (Kwon et al., 2022) | Prediction | OMFS | Direct clinical influence | CNN | OPG | 724 |
| (Kyventidis & Angelopoulos, 2021) | Diagnosis/Detection/Classification | Unspecified | Segmentation | CNN | Intra-oral Radiograph | 15254 |
| (Le et al., 2022) | Diagnosis/Detection/Classification | Orthodontics | Direct clinical influence | CNN | Lateral Cephalogram | 1293 |
| (Lee & Jeong, 2020) | Diagnosis/Detection/Classification | OMFS | Direct clinical influence | CNN | OPG, Intra-oral Radiograph | 10770 |
| (Lee et al., 2018) | Diagnosis/Detection/Classification | Cariology | Direct clinical influence | CNN | Intra-oral Radiograph | 3000 |
| (Lee, Y.H. et al., 2022) | Diagnosis/Detection/Classification | OMFS | Direct clinical influence | CNN | MRI | 2520 |
| (Lee, A. et al., 2021) | Diagnosis/Detection/Classification | Systemic Medicine | Direct clinical influence | CNN | OPG | 458 |
| (Lee,C.-T. et al., 2022) | Diagnosis/Detection/Classification | Periodontics | Direct clinical influence | CNN | OPG | 693 |
| (Lee, J.-H. et al., 2020) | Diagnosis/Detection/Classification | Oral Medicine | Direct clinical influence | CNN | OPG, CBCT | 2126 |
| (Lee, J.-H. et al., 2020a) | Prediction | Orthodontics | Direct clinical influence | CNN | Lateral Cephalogram | 400 |
| (Lee, K.-S. et al., 2020) | Diagnosis/Detection/Classification | Systemic Medicine | Direct clinical influence | CNN | OPG | 680 |
| (Lee, S.-C. et al., 2022) | Management | Orthodontics | Direct clinical influence | CNN | 3D dental scanner, CBCT | 15 |
| (Li et al., 2020) | Diagnosis/Detection/Classification | Unspecified | Segmentation | CNN | CBCT | 1160 |
| (Li, C.-W. et al., 2021) | Diagnosis/Detection/Classification | Endodontics | Direct clinical influence | CNN | Intra-oral Radiograph | 476 |
| (Li, H. et al., 2021) | Diagnosis/Detection/Classification | Periodontics | Direct clinical influence | Boosting Techniques, Object Detection using CNN | OPG | 407 |
| (Li, S. et al., 2022) | Diagnosis/Detection/Classification | Cariology, Periodontics | Direct clinical influence | CNN | Intra-oral Radiograph | 4219 |
| (Li, W. et al., 2021) | Diagnosis/Detection/Classification | Periodontics | Direct clinical influence | CNN | Phone Images | 3932 |
| (Li et al., 2023) | Diagnosis/Detection/Classification | Cariology, Periodontics | Direct clinical influence | CNN | Clinical photograph | 3696 |
| (Li, Y. et al., 2022) | Management | Endodontics | Direct clinical influence | Transformer Architecture | OPG, Intra-oral Radiograph | 245 |
| (Lian et al., 2021) | Diagnosis/Detection/Classification | Cariology | Direct clinical influence | CNN | OPG | 1160 |
| (Lim et al., 2021) | Diagnosis/Detection/Classification | OMFS | Direct clinical influence | CNN | CBCT | 138 |
| (Lin, B. et al., 2022) | Diagnosis/Detection/Classification | Orthodontics | Direct clinical influence | CNN | MRI | 9009 |
| (Lin et al., 2021) | Management | OMFS | Direct clinical influence | CNN | CBCT | 71 |
| (Lin, X. et al., 2022) | Diagnosis/Detection/Classification | Cariology | Direct clinical influence | CNN | Intra-oral Radiograph | 600 |
| (Lin, Y. et al., 2022) | Diagnosis/Detection/Classification | OMFS | Direct clinical influence | CNN | CBCT | 1036 |
| (Liu, J. et al., 2022) | Diagnosis/Detection/Classification | Paediatric Dentistry, Orthodontics | Direct clinical influence | CNN | OPG | 1580 |
| (Liu, F. et al., 2023) | Diagnosis/Detection/Classification | Endodontics, Cariology | Direct clinical influence | CNN | Intra-oral Radiograph | 188 |
| (Liu, J.L. et al., 2021) | Diagnosis/Detection/Classification | Oral Medicine | Direct clinical influence | CNN | Intra-oral Radiograph | 1023 |
| (Liu, M. et al., 2022) | Diagnosis/Detection/Classification | OMFS | Direct clinical influence | Object Detection using CNN | Intra-oral Radiograph | 1670 |
| (Liu, M.-Q. et al., 2022) | Diagnosis/Detection/Classification | OMFS | Direct clinical influence | CNN | CBCT | 254 |
| (Liu, T. et al., 2023) | Diagnosis/Detection/Classification | OMFS | Direct clinical influence | CNN | CBCT | 387 |
| (Liu, Z. et al., 2021) | Diagnosis/Detection/Classification | Oral Medicine, OMFS | Direct clinical influence | CNN | OPG | 420 |
| (Lu et al., 2023) | Diagnosis/Detection/Classification | Orthodontics, Oral Medicine, Dental Radiography | Direct clinical influence | CNN | CBCT | 150 |
| (Machado et al., 2023) | Diagnosis/Detection/Classification | OMFS | Segmentation | CNN | OPG | 437 |
| (Mahdi et al., 2020) | Diagnosis/Detection/Classification | Unspecified | Segmentation | Object Detection using CNN | OPG | 1000 |
| (Mao et al., 2021) | Diagnosis/Detection/Classification | Cariology | Direct clinical influence | CNN | Intra-oral Radiograph | 278 |
| (Mertens et al., 2021) | Diagnosis/Detection/Classification | Cariology | Direct clinical influence | CNN | Intra-oral Radiograph | 140 |
| (Miki et al., 2017) | Diagnosis/Detection/Classification | Unspecified | Segmentation | CNN | CBCT | 52 |
| (Mima et al., 2022) | Diagnosis/Detection/Classification | Unspecified | Direct clinical influence | Object Detection using CNN | OPG | 160 |
| (Min Park et al., 2021) | Prediction | Oral Medicine | Direct clinical influence | Statistical Models and Non-Deep models | MRI | 157 |
| (Mine et al., 2022) | Diagnosis/Detection/Classification | Paediatric Dentistry | Direct clinical influence | CNN | OPG | 220 |
| (Mohammad et al., 2022) | Diagnosis/Detection/Classification | Paediatric Dentistry, Orthodontics | Segmentation | CNN | OPG | 240 |
| (Moran et al., 2021) | Diagnosis/Detection/Classification | Cariology | Direct clinical influence | CNN | Intra-oral Radiograph | 592 |
| (Mori et al., 2022) | Diagnosis/Detection/Classification | Dental Radiography | Segmentation | CNN | OPG | 70 |
| (Morishita et al., 2022) | Diagnosis/Detection/Classification | Unspecified | Direct clinical influence | Object Detection using CNN | OPG | 950 |
| (Morita et al., 2023) | Diagnosis/Detection/Classification | OMFS | Segmentation | CNN | CT | 50 |
| (Mortaheb & Rezaeian, 2016) | Diagnosis/Detection/Classification | Dental Radiography | Segmentation | Statistical Models and Non-Deep models | CBCT | 14 |
| (Muhammed Sunnetci et al., 2022) | Diagnosis/Detection/Classification | Periodontics | Direct clinical influence | CNN | OPG | 1432 |
| (Muramatsu et al., 2016) | Diagnosis/Detection/Classification | Systemic Medicine | Direct clinical influence | Statistical Models and Non-Deep models | OPG | 99 |
| (Muramatsu et al., 2021) | Diagnosis/Detection/Classification | Unspecified | Direct clinical influence | CNN | OPG | 100 |
| (Murata et al., 2019) | Diagnosis/Detection/Classification | Oral Medicine | Direct clinical influence | CNN | OPG | 800 |
| Nakamoto et al. (2022) | Diagnosis/Detection/Classification | Systemic Medicine | Direct clinical influence | CNN | OPG | 1500 |
| (Ngan et al., 2016) | Diagnosis/Detection/Classification | Cariology, Endodontics, Paediatric Dentistry, Periodontics | Direct clinical influence | Statistical Models and Non-Deep models | Intra-oral Radiograph | 66 |
| (Ngoc et al., 2020) | Diagnosis/Detection/Classification | OMFS | Direct clinical influence | CNN | OPG | 447 |
| (Nishiyama et al., 2021) | Diagnosis/Detection/Classification | OMFS | Direct clinical influence | CNN | Intra-oral Radiograph | 500 |
| (Noguchi et al., 2023) | Diagnosis/Detection/Classification | Oral Medicine | Direct clinical influence | Statistical Models and Non-Deep models, Tree Based Methods | Clinical photograph | 60 |
| (Okada et al., 2015) | Diagnosis/Detection/Classification | Oral Medicine | Direct clinical influence | Tree Based Methods, Boosting Techniques | CBCT | 28 |
| (Orhan et al., 2021) | Diagnosis/Detection/Classification | OMFS | Direct clinical influence | CNN | CBCT | 130 |
| (Oztekin et al., 2023) | Diagnosis/Detection/Classification | Cariology | Direct clinical influence | CNN | OPG | 562 |
| (Paderno et al., 2021) | Diagnosis/Detection/Classification | Oral Medicine | Segmentation | CNN | Video Analysis | 226 |
| (Pan et al., 2020) | Prediction | Oral Medicine | Direct clinical influence | ANN/MLP | CT | 1386 |
| (Park, E.Y. et al., 2022) | Diagnosis/Detection/Classification | Cariology | Direct clinical influence | CNN, Object Detection using CNN | Clinical photograph | 2348 |
| (Park, H.S. et al., 2022) | Diagnosis/Detection/Classification | Dental Radiography | Segmentation | Other | CBCT | 18816 |
| (Prados-Privado et al., 2021) | Diagnosis/Detection/Classification | Dental Radiography | Direct clinical influence | CNN | OPG | 2230 |
| (Prados-Privado et al., 2021a) | Diagnosis/Detection/Classification | Unspecified | Segmentation | CNN | OPG | 8000 |
| (Preda et al., 2022) | Diagnosis/Detection/Classification | OMFS, Orthodontics | Segmentation | CNN | CBCT | 144 |
| (Qayyum et al., 2023) | Diagnosis/Detection/Classification | Cariology | Direct clinical influence | CNN | Intra-oral Radiograph | 141 |
| (Qiu et al., 2019) | Diagnosis/Detection/Classification | OMFS | Direct clinical influence | CNN | CT | 109 |
| (Qiu et al., 2021) | Diagnosis/Detection/Classification | OMFS | Segmentation | Recurrent Neural Network | CBCT | 59 |
| (Qu et al., 2022) | Prediction | Endodontics | Direct clinical influence | Tree Based Methods, Boosting Techniques | Intra-oral Radiograph, CBCT | 178 |
| (Raith et al., 2017) | Diagnosis/Detection/Classification | Unspecified | Direct clinical influence | ANN/MLP | 3D dental scanner | 129 |
| (Romeo et al., 2020) | Prediction | Oral Medicine | Direct clinical influence | Tree Based Methods, ANN/MLP, Statistical Models and Non-Deep models, Boosting Techniques | CT | 40 |
| (Ryu et al., 2023) | Management | Orthodontics | Direct clinical influence | CNN | Clinical photograph | 3136 |
| (Saïd et al., 2020) | Diagnosis/Detection/Classification | OMFS | Direct clinical influence | CNN | Intra-oral Radiograph, OPG | 1206 |
| (Schlickenrieder et al., 2021) | Diagnosis/Detection/Classification | Cariology | Direct clinical influence | CNN | Clinical photograph | 2352 |
| (Schönewolf et al., 2022) | Diagnosis/Detection/Classification | Paediatric Dentistry | Direct clinical influence | CNN | Clinical photograph | 3241 |
| (Schwendicke et al., 2022b) | Management | Cariology | Direct clinical influence | CNN | Intra-oral Radiograph | 23 |
| (Seo et al., 2021) | Diagnosis/Detection/Classification | Orthodontics | Direct clinical influence | CNN | CBCT | 600 |
| (Shahnavazi & Mohamadrahimi, 2023) | Diagnosis/Detection/Classification | OMFS | Direct clinical influence | ANN/MLP | OPG | 190 |
| (Shin et al., 2021) | Prediction | Orthodontics | Direct clinical influence | CNN | Cephalogram | 413 |
| (Singh et al., 2021) | Diagnosis/Detection/Classification | Systemic Medicine | Direct clinical influence | CNN | OPG | 70 |
| (Son et al., 2021) | Diagnosis/Detection/Classification | OMFS | Direct clinical influence | CNN | Intra-oral Radiograph | 420 |
| (Song et al., 2022) | Diagnosis/Detection/Classification | Oral Medicine | Direct clinical influence | CNN | Clinical photograph | 492 |
| (Song et al., 2018) | Diagnosis/Detection/Classification | Oral Medicine | Direct clinical influence | CNN | Clinical photograph | 170 |
| (Song et al., 2023) | Diagnosis/Detection/Classification | Oral Medicine | Direct clinical influence | CNN | Clinical photograph | 3040 |
| (Sukegawa et al., 2020) | Diagnosis/Detection/Classification | OMFS | Direct clinical influence | CNN | OPG | 8859 |
| (Sukegawa et al., 2022) | Diagnosis/Detection/Classification | Systemic Medicine | Direct clinical influence | CNN | OPG | 778 |
| (Sukegawa et al., 2022a) | Diagnosis/Detection/Classification | Orthodontics | Direct clinical influence | CNN | OPG | 1330 |
| (Sukegawa et al., 2022b) | Diagnosis/Detection/Classification | OMFS | Direct clinical influence | CNN | OPG | 10191 |
| (Sun et al., 2022) | Prediction | Oral Medicine | Direct clinical influence | CNN | CT | 3817 |
| (Takahashi et al., 2021) | Diagnosis/Detection/Classification | Prosthodontics | Direct clinical influence | CNN | Clinical photograph | 1184 |
| (Takebe et al., 2022) | Diagnosis/Detection/Classification | OMFS | Direct clinical influence | CNN | OPG | 518 |
| (Tao & Wang, 2022) | Diagnosis/Detection/Classification | Dental Radiography | Segmentation | CNN | CT | 1500 |
| (Tareq et al., 2023) | Diagnosis/Detection/Classification | Cariology | Direct clinical influence | CNN, Object Detection using CNN | Clinical photograph | 1703 |
| (Ter Horst et al., 2021) | Prediction | OMFS | Direct clinical influence | ANN/MLP | CBCT | 133 |
| (Tian et al., 2022) | Management | Prosthodontics | Direct clinical influence | CNN, GANs | 3D dental scanner | 1000 |
| (Tobias et al., 2022) | Diagnosis/Detection/Classification | Oral Medicine | Direct clinical influence | CNN | Clinical photograph | 1636 |
| (Tomita et al., 2021) | Diagnosis/Detection/Classification | Oral Medicine | Direct clinical influence | Statistical Models and Non-Deep models | CBCT | 23 |
| (Tomita et al., 2021a) | Diagnosis/Detection/Classification | Oral Medicine | Direct clinical influence | CNN | CT | 320 |
| (Verhelst et al., 2021) | Diagnosis/Detection/Classification | Unspecified | Segmentation | CNN | CBCT | 160 |
| (Vila-Blanco et al., 2021) | Diagnosis/Detection/Classification | Orthodontics, Prosthodontics, OMFS | Direct clinical influence | CNN | OPG | 1195 |
| (Vinayahalingam et al., 2023) | Diagnosis/Detection/Classification | OMFS | Segmentation | CNN | CBCT | 154 |
| (Vinayahalingam et al., 2021) | Diagnosis/Detection/Classification | OMFS | Direct clinical influence | CNN | OPG | 81 |
| (Vinayahalingam et al., 2019) | Diagnosis/Detection/Classification | Cariology | Direct clinical influence | CNN | Intra-oral Radiograph | 400 |
| (Vinayahalingam et al., 2022) | Diagnosis/Detection/Classification | OMFS | Direct clinical influence | CNN, Transformer Architecture, Object Detection using CNN, Transformer Architecture | OPG | 6404 |
| (Vollmer et al., 2022) | Prediction | OMFS | Direct clinical influence | CNN | Intra-oral Radiograph | 300 |
| (Vranckx et al., 2020) | Prediction | OMFS | Direct clinical influence | CNN | OPG | 838 |
| (Wang et al., 2022) | Diagnosis/Detection/Classification | OMFS | Direct clinical influence | CNN | CT | 686 |
| (Wang et al., 2020) | Diagnosis/Detection/Classification | Cariology | Direct clinical influence | CNN | Clinical photograph | 7200 |
| (Wang et al., 2023) | Diagnosis/Detection/Classification | Cariology | Direct clinical influence | CNN | Clinical photograph | 83 |
| (Wang et al., 2021) | Diagnosis/Detection/Classification | Oral Medicine | Segmentation | CNN | CBCT | 90 |
| (Wang et al., 2022a) | Prediction | OMFS, Orthodontics | Direct clinical influence | Boosting Techniques, Tree Based Methods, Statistical Models and Non-Deep models | Lateral Cephalogram | 108 |
| (Warin et al., 2021) | Diagnosis/Detection/Classification | Oral Medicine | Direct clinical influence | CNN, Object Detection using CNN | Clinical photograph | 700 |
| (Warin et al., 2022) | Diagnosis/Detection/Classification | OMFS | Direct clinical influence | CNN | Intra-oral Radiograph | 1710 |
| (Warin et al., 2022b) | Diagnosis/Detection/Classification | Endodontics | Direct clinical influence | CNN | Intra-oral Radiograph | 200 |
| (Warin et al., 2022a) | Diagnosis/Detection/Classification | Oral Medicine | Direct clinical influence | CNN, Object Detection using CNN | Clinical photograph | 980 |
| (Watanabe et al., 2021) | Diagnosis/Detection/Classification | OMFS, Oral Medicine | Direct clinical influence | CNN | OPG | 412 |
| (Welch et al., 2020) | Diagnosis/Detection/Classification | Oral Medicine | Segmentation | CNN | CT | 1538 |
| (Welch et al., 2020) | Diagnosis/Detection/Classification | Dental Radiography | Direct clinical influence | CNN | CT | 2112 |
| (Widyaningrum et al., 2023) | Diagnosis/Detection/Classification | Oral Medicine | Direct clinical influence | Statistical Models and Non-Deep models | Intra-oral Radiograph | 102 |
| (Wu, C. et al., 2022) | Diagnosis/Detection/Classification | Oral Medicine | Direct clinical influence | CNN | CT | 114 |
| (Wu, T.H. et al., 2022) | Diagnosis/Detection/Classification | Orthodontics | Segmentation | CNN | 3D dental scanner | 35 |
| (Xia et al., 2017) | Diagnosis/Detection/Classification | Unspecified | Segmentation | Other | CT, CBCT | 10 |
| (Xiao et al., 2022) | Diagnosis/Detection/Classification | OMFS | Direct clinical influence | CNN | CBCT | 70 |
| (Xie et al., 2022) | Diagnosis/Detection/Classification | Orthodontics | Direct clinical influence | Statistical Models and Non-Deep models | CBCT | 231 |
| (Xu et al., 2019) | Management | Orthodontics | Segmentation | CNN | CT | 1200 |
| (Xu et al., 2021) | Diagnosis/Detection/Classification | Oral Medicine, Prosthodontics | Segmentation | CNN | CT | 60 |
| (Xu et al., 2021a) | Management | OMFS | Segmentation | CNN | CT | 270 |
| (Xu et al., 2023) | Diagnosis/Detection/Classification | Oral Medicine | Direct clinical influence | Object Detection using CNN | CECT | 5412 |
| (Yüksel et al., 2021) | Diagnosis/Detection/Classification | Unspecified | Direct clinical influence | CNN, Statistical Models and Non-Deep models | OPG | 1005 |
| (Yadollahi et al., 2015) | Diagnosis/Detection/Classification | Orthodontics | Segmentation | Unspecified | Clinical photograph |  |
| (Yang et al., 2020) | Diagnosis/Detection/Classification | Oral Medicine | Direct clinical influence | CNN | OPG | 1603 |
| (Yang et al., 2023) | Diagnosis/Detection/Classification | Endodontics | Direct clinical influence | CNN | CBCT | 1641 |
| (Yang et al., 2022) | Prediction | Endodontics | Direct clinical influence | CNN | Intra-oral Radiograph, OPG | 372 |
| (Yao et al., 2022) | Diagnosis/Detection/Classification | Orthodontics | Direct clinical influence | CNN | Lateral Cephalogram | 512 |
| (Yaren Tekin et al., 2022) | Diagnosis/Detection/Classification | Unspecified | Segmentation | Object Detection using CNN | Intra-oral Radiograph | 300 |
| (Yesiltepe et al., 2022) | Diagnosis/Detection/Classification | Oral Medicine | Direct clinical influence | Object Detection using CNN | OPG | 493 |
| (You et al., 2020) | Diagnosis/Detection/Classification | Cariology, Periodontics | Direct clinical influence | CNN | Clinical photograph | 886 |
| (Yu et al., 2022) | Diagnosis/Detection/Classification | Oral Medicine | Direct clinical influence | CNN | OPG | 287 |
| (Zadrożny et al., 2022) | Diagnosis/Detection/Classification | Dental Radiography | Direct clinical influence | CNN | OPG | 30 |
| (Zhang, C. et al., 2023) | Prediction | OMFS | Direct clinical influence | CNN | Intra-oral Radiograph, OPG | 1080 |
| (Zhang, J. N. et al., 2023) | Diagnosis/Detection/Classification | Orthodontics | Direct clinical influence | CNN | Lateral Cephalogram | 256 |
| (Zhang et al., 2022) | Diagnosis/Detection/Classification | Cariology | Direct clinical influence | CNN | Clinical photograph | 3932 |
| (Zhao et al., 2021) | Diagnosis/Detection/Classification | Oral Medicine | Direct clinical influence | CNN | Lateral Cephalogram | 581 |
| (Zhong et al., 2022) | Diagnosis/Detection/Classification | Oral Medicine | Segmentation | ANN/MLP | CT | 1673 |
| (Zhou et al., 2022) | Diagnosis/Detection/Classification | Cariology, Paediatric Dentistry | Direct clinical influence | CNN | OPG | 210 |
| (Zhu et al., 2022) | Diagnosis/Detection/Classification | Paediatric Dentistry | Direct clinical influence | CNN | Intra-oral Radiograph | 438 |
| (Zhu et al., 2021) | Management | OMFS | Direct clinical influence | Object Detection using CNN | OPG | 503 |
